# Effects of long-term individual housing of middle-aged female *Octodon degus* on spatial learning and memory in the Barnes maze task

**DOI:** 10.3389/fnbeh.2023.1221090

**Published:** 2023-08-03

**Authors:** Natalija Popović, Beatriz Baño-Otalora, María Ángeles Rol, César Venero, Juan Antonio Madrid, Miroljub Popović

**Affiliations:** ^1^Department of Human Anatomy and Psychobiology, Faculty of Medicine, University of Murcia, Murcia, Spain; ^2^Biomedical Research Institute of Murcia, Virgen de la Arrixaca University Hospital, University of Murcia, Murcia, Spain; ^3^Chronobiology Lab, Department of Physiology, Faculty of Biology, University of Murcia, Mare Nostrum Campus, Instituto Universitario de Investigación en Envejecimiento, Murcia, Spain; ^4^Ciber Fragilidad y Envejecimiento Saludable, Madrid, Spain; ^5^Department of Psychobiology, Universidad Nacional de Educación a Distancia, Madrid, Spain; ^6^Instituto Mixto de Investigación–Escuela Nacional de Sanidad, Madrid, Spain

**Keywords:** Barnes maze, light-dark test, *Octodon degus*, social isolation, anxiety, learning and memory

## Abstract

**Introduction:**

Prolonged social isolation is a form of passive chronic stress that has consequences on human and animal behavior. The present study was undertaken to elucidate whether the long-term isolation would precipitate age-related changes in anxiety and spatial learning and memory in degus.

**Methods:**

We investigated the effects of long-term social isolation on anxiety levels in the light-dark test, and spatial orientation abilities in the Barnes maze. Middle-aged female *Octodon degus* were allocated to either group-housed (3 animals per cage) or individually-housed for 5 months.

**Results:**

Under this experimental condition, there were no significant group differences in the anxiety level tested in the light-dark test and in the motivation to escape from the Barnes maze. There were no significant differences in cortisol levels between individually- and group-housed animals. On the last acquisition training day of spatial learning, individually- housed animals had a significantly higher number of correct responses and a smaller number of reference and working memory errors than the group-housed animals. In addition, isolated animals showed a tendency for reference and working memory impairment on the retention trial, while group-housed degus showed improvement in these parameters.

**Discussion and conclusion:**

The present study indicates that prolonged social isolation during adulthood in female degus has a dual effect on spatial orientation. Specifically, it results in a significant improvement in acquisition skills but a slight impairment in memory retention. The obtained cognitive changes were not accompanied by modification in anxiety and cortisol levels.

## 1. Introduction

Sustained lack of social support and chronic social isolation are considered stressful situations that can have deleterious consequences on animal and human health (Cacioppo et al., 2010; Cruces et al., 2014; Holt-Lunstad et al., 2015; Smith et al., 2018). Social isolation of adults or older adults commonly involves the reduction or removal of individuals from already established and accustomed social interactions. Only a few studies have examined the effects of social isolation on the behavior of adult and older individuals. It has been shown that social isolation results in a state of emotional numbness (Twenge et al., 2003) and anticipated loneliness and poor performance on tasks assessing cognitive functions (Baumeister et al., 2002). Longitudinal studies of cognitive functioning performed on older adults have reported that loneliness and social isolation predict age-related cognitive decline and risk for Alzheimer’s disease (Wilson et al., 2007; Yu et al., 2021; Yorgason et al., 2022), while perceived social bonding and interpersonal synchrony display beneficial effects on mental and physical health (Fratiglioni et al., 2004; Cacioppo and Cacioppo, 2012).

Strong social bonds from early infancy through the life course of the long-lived diurnal caviomorph, *Octodon degus*, (Fulk, 1976; Ebensperger et al., 2004; Hayes et al., 2009), bring this species into focus as a relevant rodent model to study the effect of social isolation. In the last decades, the impact of early separation stress on brain development and behavior of the offspring has been extensively documented in this species. For instance, maternal separation and social isolation altered the balance between dopaminergic and serotonergic immunoreactive fiber innervation or receptor expression in several brain regions, including the medial prefrontal cortex (Braun et al., 2000, 2013; Poeggel et al., 2003; Ziabreva et al., 2003a) and hippocampal areas (Ziabreva et al., 2003b; Gos et al., 2006).

The 8-day-old degus, exposed to parental separation, exhibited increased running activity and rearing behavior in the open-field test (Braun et al., 2003). The 3-week-old degus subjected daily to 1 h separation from the parents showed similar results as controls, for time spent and the number of entries in the five compartments of the elevated plus-maze, but significantly more rearing along the walls of the closed arms of the maze, indicating reduced anxiety levels (Becker et al., 2007). Pups reared in isolation showed a significantly stronger preference for sucrose intake over water than partially isolated and socially housed degus (Colonnello et al., 2011). They also exhibited increased risk-taking behavior in fearful situations, spending less time freezing in the startle test and searching more in the open field and novelty tests (Colonnello et al., 2011). Interestingly, stress inoculation for 3 weeks, performed by 1 h daily separation of pups from parents starting at the day of birth, improved two-way avoidance learning in 3-week- and 90-day-old degus, quantitatively and qualitatively depending on applied foot-shock intensity (Abraham and Gruss, 2010). A recent study demonstrated that social isolation of degus from the post-natal and post-weaning period until adulthood impaired spatial memory in the Barnes maze test (Rivera et al., 2020).

Our previous study pointed out that long-term isolation of middle-aged female degus impaired contextual, but not cued fear conditioning, reduced hippocampal levels of polysialylated form of neural cell adhesion molecule (PSA-NCAM) in the hippocampus and produced shrinkage of its CA1 subfield (Pereda-Pérez et al., 2013). The present study was undertaken to elucidate whether the long-term isolation would precipitate age-related changes in anxiety and spatial learning and memory in degus.

## 2. Materials and methods

### 2.1. Animals

The experiments were performed on eighteen female *Octodon degus* at the age of 39–44 months. The animals were obtained from the Animal Service of the University of Murcia and initially, they were housed in groups of three, in transparent Plexiglas cages (48.3 cm length × 26.7 cm width × 20.3 cm height) and under standard conditions: lights on from 08:00 to 20:00 h, controlled temperature of 22 ± 1°C and 60% humidity. The degus were fed *ad libitum* using a commercial rat chow (A04 rat–mouse maintenance Panlab).

### 2.2. Experimental procedure

The experiments were performed from 09:00 to 15:00 h and video recorded to enable subsequent evaluation. The experimental equipment was cleaned with 70% ethanol before each animal was tested. Given the significance of standardizing testing procedures (Commins and Kirby, 2019), the behavioral tests applied in the present study were conducted following established protocols in our laboratory. In all experiments, the experimenter carefully moved to the adjacent laboratory after placing the animal in the testing equipment, aiming to minimize any potential impact on animal behavior and maintain control of the experiment through the computer screen.

#### 2.2.1. Experimental groups and design

**FIGURE 1:**
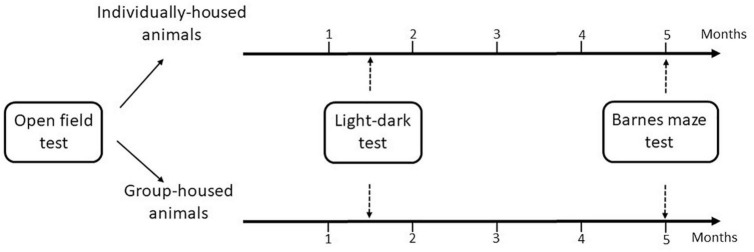
Flow diagram of the experimental design.

Initially, according to the behavior in the open field (a test used to evaluate anxiety/exploration-like and habituation levels), the animals were divided into two homogenous groups: individually-housed and group-housed. In addition, there was no significant difference in body weight between the individually-housed group (228.68 ± 7.67 g) and the group-housed (237.69 ± 8.82 g) group. The individual cages were separated by cardboard panels to avoid visual contact. After 1 month and a half of maintenance in these conditions, the anxiety levels of the animals were assessed in the light-dark test. Individually-housed animals were kept socially isolated for the following 3 months and a half. The group-housed animals continued to be housed in 3/cage for the entire experimental time. Then, the cognitive abilities of animals were determined using the Barnes maze test. All behavioral tests were performed in the diestrous phase of the oestrous cycle. Figure 1 is a schematic diagram of behavior testing time-course for the two experimental groups.

#### 2.2.2. Open field habituation test (evaluation of baseline anxiety and cognitive level)

The open field test was performed in a square white plywood box (100 cm width × 100 cm length × 40 cm height) which was located in the center of the room with white walls. The floor of the open-field box was divided into 16 sidewall-outer and 9 inner squares (each 20 cm × 20 cm). A camera-supported stick was placed 30 cm away from one side of the open-field box and the camera was positioned 2.4 m above the center of the open field. On day 1 (acquisition trial), the animals were initially placed in the center of the open field and their behavior was video recorded for 5 min (Popović et al., 2009). After that, the degus were returned to their home cage. The same procedure was repeated during the retention trial (48 h after the acquisition trial). At both acquisition and retention trials, the open-field test was performed under 300 lux light intensity. In the open field test, the following parameters were recorded: ambulation along the sidewall and in the central area (number of outer and inner squares entered, respectively), the number of rearings (standing on the hind legs, with or without contact with the sides of the box) and the number of fecal boli deposition.

With the aim to correct the individual baseline differences in the studied parameters, we used a change ratio score to compare behavior during the acquisition and retention trials. This score is calculated according to criteria established by Bolivar (2009): day 2 parameter value/(day 1 parameter value + day 2 parameter value). Thus, if the change ratio score approaches 0.5, no change in behavior has occurred (i.e., no habituation). However, if the value tends to 0 there is evidence of habituation (Popović et al., 2017; Poveda et al., 2020).

#### 2.2.3. Light-dark test

A cage made of Perspex sheets was used for this test, divided into two equally sized sections (21 cm width × 21 cm length × 21 cm height): white and black. The white section was illuminated by a 24 V–10 W bulb, providing a floor-level illumination of 180 lx. The black (dark) section was covered by a lightproof lid. There was no appreciable illumination in the dark section. Both sections were connected by a 7 cm × 7 cm opening at the floor level. The animal was placed in the light section facing away from the entrance to the dark section and allowed to explore the apparatus freely for 5 min (Popović et al., 2009). The following parameters were recorded during the test: latency to the first enter the dark compartment with all four feet, time spent in the light compartment, number of the light-dark transitions with all four feet, number of risk assessment behavior of the light and dark compartment (head and/or incomplete body dipping into either one compartment) and number of fecal boli deposition.

#### 2.2.4. Barnes maze test

The Barnes maze consisted of a circular white Plexiglas platform (160 cm in diameter) with 18 circular holes and surrounded by 45 cm high wall (for details, please see Popović et al., 2010). All holes, except the target one, were blocked with mesh. A plastic transparent escape cage, filled with the bedding from the home cage, was positioned under the escape hole. The start box was a white, open-ended cylinder easily lifted from the platform to the roof, approximately 3 m above. The maze was positioned in a room with many extra-maze cues (e.g., geometric figures, pictures, cage racks, furniture, etc.) to permit the orientation of degu in the space. The Barnes maze platform was uniformly illuminated (300 lx) by fluorescent lights located on the ceiling. A web camera was secured on the roof, directly above and focused on the maze surface, and plugged directly into the data acquisition computer. This allowed the experimenter, while seated outside of the field of view of degu, to raise the start box, and immediately begin recording of its behavior.

The procedure was divided into 3 phases: habituation trial, acquisition trials and retention trial (Popović et al., 2010). The habituation session began with placing the animal into the transparent escape box (filled with the bedding from the home cage) for 2 min. Subsequently, the animal was placed near the escape hole surrounded by the start box and given 1 min to escape. If the animal did not enter the escape box, within that time, it was carefully lifted and guided through the target hole into the escape box. The animal was left in the escape box for 2 min. Finally, the animal was placed in the center of the maze, and left undisturbed for the following 4 min allowing it the opportunity to enter the escape box. If the animal failed to enter the escape box during the allotted time, it was placed into the escape box manually, following the procedure mentioned earlier, and left there for 2 min. Each part of the habituation session was separated by a 5 min resting phase, during which *degus* remained in its home cage.

Two days after the habituation session, the animals were trained for 5 days and then exposed to a retrieval session, 7 days later. Each training or retrieval session consisted of four consecutive 4.5 min trials, separated by a 5 min resting phase in the animal home cage. At the beginning of each trial, each degus was confined for 30 s in the start box in the center of the maze until a trial was initiated by lifting the cylinder, followed by free maze exploration for the 4-min. If the degus did not enter the escape box within the allotted time, it was gently picked up and placed into the escape box. The animal was left in the escape box for 2 min before being returned to its home cage for 5 min. The escape hole remained at a constant position throughout all trials and sessions. The following parameters were recorded (for details please see Popović et al., 2010): latency to the first visit of the escape hole, latency to escape, number of correct responses, number of reference memory errors to the first visit of the escape hole (in further text number of reference memory errors) and number of working memory errors to the first visit of escape hole (in further text number of working memory errors).

#### 2.2.5. Blood sampling procedure and plasma cortisol measurement

Animals were sacrificed by rapid decapitation, and trunk blood was collected into sodium-heparinized tubes to prevent coagulation and centrifuged for 15 min at 4°C to 2,026 *g*. The plasma obtained was stored at −80°C until cortisol analysis was carried out. Plasma cortisol was measured using an ELISA (DRG Instruments GmbH, Germany) with a sensitivity of 2.5 ng/mL.

### 2.3. Statistical analysis

Four animals were excluded from statistical analysis due to developed cataract. Finally, each group was composed of seven animals.

The data are presented as mean ± standard error of the mean (SEM). The statistical analysis was performed using the SPSS 24.0 statistical package (IBM Corp., Armonk, NY, USA). The Shapiro–Wilk test was used to determine the distribution of data values. In order to normalize some data distribution (latency to escape, number of correct responses, and number of working memory errors) the natural logarithmic transformation was applied before the between-group statistical analysis was performed.

The acquisition of spatial learning in the Barnes maze test was first analyzed with the general linear model repeated measures analysis (GLM) followed by two-tailed Student’s *t*-test for independent samples. A two-tailed Student’s *t*-test for independent samples was also used to compare groups in the open-field, light-dark test, the retention trial of the Barnes maze, and cortisol level. A two-tailed Student’s *t*-test for paired samples was used to compare the data in each tested group between the last acquisition day and retention day in the Barnes maze, as well as between the acquisition and retention trial in the open field test. Differences were considered statistically significant if *p* < 0.05.

## 3. Results

### 3.1. Open field habituation test (baseline anxiety and cognitive level)

The detailed outcome of the statistical analysis of the data obtained in the open-field habituation test is presented in Table 1.

**TABLE 1:** Statistical analyses performed in the open field habituation test.

Parameter	Two-tailed Student’s *t*-test for independent samples (degrees of freedom = 12)			Two-tailed Student’s *t*-test for paired samples (degrees of freedom = 6)	
	**Individual vs. group housed**			**Acquisition vs. 48 h retention trial**	
	**Acquisition trial**	**48 h retention trial**	**Score**	**Individual-housed**	**Group-housed**
No. of outer squares entered	*t* = 0.426, *p* = 0.678	*t* = 1.289, *p* = 0.222	*t* = 0.616, *p* = 0.550	***t* = 3.627,** ***p* = 0.011**	***t* = 3.622,** ***p* = 0.011**
No. of inner squares entered	*t* = −0.279, *p* = 0.785	*t* = 0.064, *p* = 0.950	*t* = 0.350, *p* = 0.732	*t* = −0.596, *p* = 0.573	*t* = −0.326, *p* = 0.755
No. rearing	*t* = 0.408, *p* = 0.691	*t* = 0.592, *p* = 0.565	*t* = 0.213, *p* = 0.835	*t* = 1.469, *p* = 0.192	*t* = 1.637, *p* = 0.153
No. fecal boli deposited	*t* = 0.612, *p* = 0.552	*t* = −0.153, *p* = 0.881	*t* = −1.606, *p* = 0.134	*t* = 1.000, *p* = 0.356	*t* = −1.549, *p* = 0.172

The bold values indicate the statistically significant differences between groups.

**FIGURE 2:**
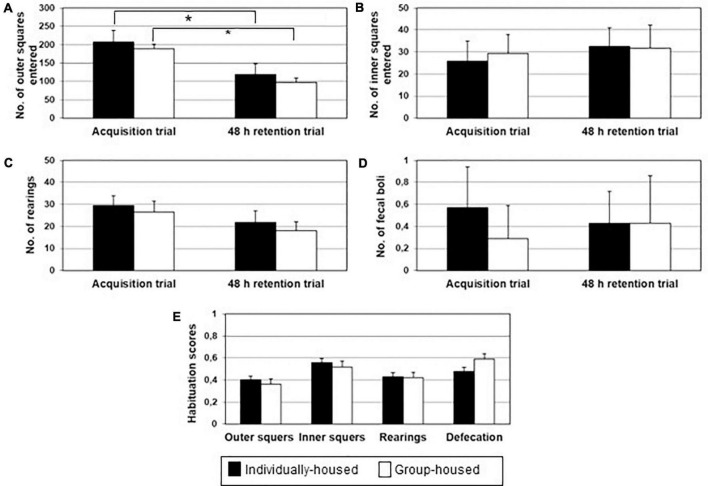
Ambulation in the board area **(A)**, ambulation in the central area **(B)**, number of rearing **(C)**, defecation **(D)** and open field habituation score **(E)** in individually- (black bars) and group-housed (clear bars) female degus, in the open field task. The data are presented as mean ± standard error of the mean (SEM). *p < 0.05 vs. acquisition trial.

There were no significant group differences during the acquisition and retention trial in the ambulation along the sidewall and in the central area, the number of rearings and the number of fecal boli deposition. Furthermore, no significant differences were found between groups in habituation scores for ambulation along the sidewall and in the central area, the number of rearings and number of fecal boli deposition. Nevertheless, a two-tailed Student’s *t*-test for paired-samples demonstrated that ambulation along the sidewall was significantly lower on 48 h retention trial in comparison to the acquisition trial in both, individually- and group-housed degus (*p* < 0.05) (Figure 2A), indicating a certain habituation in both groups. There were no significant differences in other tested parameters between acquisition and the 48 h retention trial: ambulation in the central area (Figure 2B), number of rearings (Figure 2C) and number of fecal boli deposition (Figure 2D).

### 3.1. Light-dark test

**FIGURE 3:**
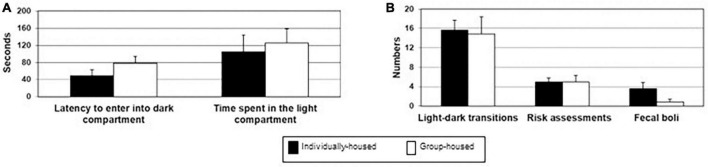
Latency to enter into the dark box and total time spent in the light compartment **(A)**, number of light-dark transitions, number of risk assessments and number of defecation boli **(B)** in individually- (black bars) and group-housed female degus (clear bars), in the light-dark test. The data are presented as mean ± standard error of the mean (SEM).

There were no significant differences between individually- and group-housed animals in their latency to enter the dark part of the box (*t*_12_ = 0.122, *p* = 0.905; Figure 3A), total duration in the light box (*t*_12_ = −0.564, *p* = 0.583; Figure 3A), the total number of transitions (*t*_12_ = 0.213, *p* = 0.835; Figure 3B), the number of risk assessments (*t*_12_ = 0.000, *p* = 1.000; Figure 3B) and the number of fecal boli deposition (*t*_12_ = 1.734, *p* = 0.109; Figure 3B).

### 3.2. Barnes maze test

The detailed outcome of the statistical analysis of the data obtained in the Barnes maze test is presented in Table 2.

**TABLE 2:** Statistical analyses performed in the Barnes maze test.

Parameter	General linear model repeated measures analysis (degrees of freedom = 1)			Two-tailed Student’s *t*-test for independent samples (degrees of freedom = 12)						Two-tailed Student’s *t*-test for paired samples (degrees of freedom = 6)	
	**Effect of training**	**Effect of group**	**Interaction effect of training and group**	**Individual vs. group-housed**						**Last acquisition day vs. retention trial**	
				**Day 1**	**Day 2**	**Day 3**	**Day 4**	**Day 5**	**Retention trial**	**Individual-housed**	**Group-housed**
Latency to the first visit of escape hole	***F* = 29.585**, ***p* = 0.001**	*F* = 0.428, *p* = 0.525	*F* = 0.622, *p* = 0.446	*t* = 0.405, *p* = 0.693	*t* = −0.905, *p* = 0.383	*t* = 0.543, *p* = 0.597	*t* = −0.721, *p* = 0.485	*t* = −1.731, *p* = 0.109	*t* = −0.113, *p* = 0.912	*t* = −0.664, *p* = 0.531	*t* = 1.680, *p* = 0.144
Latency to escape	***F* = 118.27**, ***p* = 0.001**	*F* = 0.218, *p* = 0.649	***F* = 9.656**, ***p* = 0.009**	*t* = 1.290, *p* = 0.221	*t* = −0.368, *p* = 0.719	*t* = −0.593, *p* = 0.564	*t* = −0.015, *p* = 0.988	*t* = −1.780, *p* = 0.100	*t* = −0.521, *p* = 0.612	*t* = 0.167, *p* = 0.873	*t* = 1.618, *p* = 0.157
No. correct responses	*F* = 2.979, *p* = 0.110	***F* = 5.696**, ***p* = 0.034**	*F* = 2.979, *p* = 0.110	*t* = 1.000, *p* = 0.337	*t* = 0.612, *p* = 0.552	*t* = 0.794, *p* = 0.443	*t* = 0.794, *p* = 0.443	***t* = 3.285**, ***p* = 0.007**	*t* = 0.187, *p* = 0.855	*t* = 1.411, *p* = 0.208	*t* = −1.441, *p* = 0.200
No. reference memory errors	***F* = 13.447**, ***p* = 0.003**	***F* = 4.806**, ***p* = 0.049**	*F* = 0.176, *p* = 0.682	*t* = −0.397, *p* = 0.699	*t* = −1.856, *p* = 0.088	*t* = −1.407, *p* = 0.185	*t* = −0.512, *p* = 0.618	***t* = −2.261**, ***p* = 0.043**	*t* = −0.298, *p* = 0.771	*t* = −0.943, *p* = 0.382	*t* = 1.658, *p* = 0.148
No. working memory errors	***F* = 15.610**, ***p* = 0.002**	*F* = 2.066, *p* = 0.176	*F* = 0.802, *p* = 0.388	*t* = 0.288, *p* = 0.779	*t* = −1.732, *p* = 0.109	*t* = −0.591, *p* = 0.565	*t* = −0.115, *p* = 0.910	***t* = 2.174**, ***p* = 0.050**	*t* = −0.734, *p* = 0.477	***t* = −2.801**, ***p* = 0.031**	*t* = 0.085, *p* = 0.935

The bold values indicate the statistically significant differences between groups.

#### 3.2.1. Acquisition period

The GLM showed a significant effect of training on latency to the first visit of the escape hole (*p* < 0.001), latency to escape (*p* < 0.001), the number of reference memory errors (*p* < 0.01), and number of working memory errors (*p* < 0.01), but not on the number of correct responses.

The same analysis showed a significant effect of the group on the number of correct responses (*p* < 0.05) and the number of reference memory errors (*p* < 0.05), but not on latency to the first visit of the escape hole, latency to escape and the number of working memory errors.

The interaction effect of training and group was significant for latency to escape (*p* < 0.01). There was no significant effect of the interaction of training and group on latency to the first visit of the escape hole, number of correct responses, number of reference memory errors, and number of working memory errors.

**FIGURE 4:**
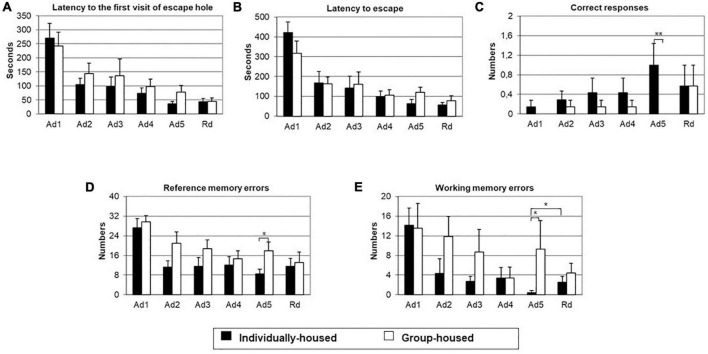
Latency to the first visit of escape hole **(A)**, latency to escape **(B)**, number of correct responses **(C)**, number of reference memory errors to the first visit of escape hole **(D)** and number of working memory errors to the first visit of escape hole **(E)** in individually- (black bars) and group-housed (clear bars) female degus exposed to Barnes maze test. The data are presented as mean ± standard error of the mean (SEM). *p < 0.05; **p < 0.01 vs. individual-housed female degus.

Two-tailed Student’s *t*-test for independent samples demonstrated that, on the last training day, individually-housed animals had significantly a higher number of correct responses (*p* < 0.01) and significantly a smaller number of reference memory errors (*p* < 0.05) and number of working memory errors (*p* < 0.05) than group-housed animals (Figures 4C, D, E, respectively).

#### 3.2.2. Retention trial

On the retention day (Figure 4), there were no group differences in measured parameters: latency to the first visit of the escape hole, latency to escape, number of correct responses, number of reference memory errors, and number of working memory errors. In comparison to the last acquisition day, individually-housed animals showed a significant increase in the number of working memory errors (*p* < 0.05). Moreover, in this group, existed a non-significant tendency for an increase in the number of reference memory errors and a decrease in the number of correct responses on the retention trial. On the other hand, group-housed animals showed non-significant tendency for a decrease of reference memory errors and working memory errors and an increase in the number of correct responses on the retention trial.

### 3.3. Cortisol level

There were no significant differences in cortisol levels between individually- (305.86 ± 85.74) and group-housed animals (468.12 ± 143.84) (*t*_10_ = −0.969, *p* = 0.355).

## 4. Discussion

In the present study, in order to create two well-balanced groups with similar anxiety and cognitive level, the open field test was performed before the initiation of the social isolation procedure. Even though it was expected that long-term social isolation would have an impact on anxiety levels and cognition, we only found that applied isolation protocol significantly improved acquisition and slightly impaired retention of spatial memory in middle-aged female degus in the Barnes maze task.

The anxiety level was evaluated by light-dark test and spatial navigation in an 18-hole Barnes circular maze, as a moderate-stressful and non-food-motivated test (Gawel et al., 2019). Based on the obtained results, long-term social isolation of middle-aged female degus did not have a significant effect on their anxiety-like behavior in the light-dark test or their motivation and speed in finding the escape hole in the Barnes maze task. These findings suggest that their anxiety levels were not affected by being chronically housed alone. Similarly, a previous study indicated that 6 weeks of social isolation did not affect depression/anxiety-like behavior in 8-month-old Sprague-Dawley male rats (Ren et al., 2015). However, 18-month-old female mice, when kept socially isolated for 6 months, exhibited increased neophobia and anxiety in the corner and in the open-field tests (Arranz et al., 2009).

The individual-housed female degus completed the acquisition of spatial learning with a higher number of correct responses and a lower number of reference and working memory errors than group-housed animals. Comparing the last training day with the retention trial, group-housed degus showed a tendency for memory improvement. In contrast, individually-housed animals showed a tendency for memory impairment. Like the present data, 18-month-old guinea pigs housed individually for 2 weeks outperformed cohabitating peers showing a more efficient encoding of spatial information, with significantly decreased latency time and error rate over a 5 days acquisition phase of the labyrinth task (Machatschke et al., 2011). Moreover, in this study, there were no significant differences between paired and single-housed animals in the retention test performed 3 days after acquisition. In contrast, several studies demonstrated that social isolation of middle-aged, as well as aged mice and rats induced memory impairment in spatial navigation tasks such as the Morris water maze (Arranz et al., 2009; Ren et al., 2015; Wang et al., 2018; Pereda-Pérez et al., 2019) and Y maze (Doulames et al., 2014; Wang et al., 2018). In addition, in 17-month-old aged male APP/PS1 double mutant transgenic mice, 3 months of social isolation exacerbated the memory impairment in the Y and Morris water maze tasks and diminished the exploration in the center of the open field (Huang et al., 2015).

In general, the behavioral effects of social isolation are determined by several factors such as duration of isolation housing, species, sex, age of the experimental rodents (either during isolation or testing) as well as the selected behavioral tests. Most of the behavioral studies testing the effect of social deprivation have been performed during the juvenile period, post-pubertal period, or in young adult animals (Arakawa, 2018). Relatively few studies have been dedicated to examining the effect of social isolation when onset occurs in middle-aged and aged rodents (Arranz et al., 2009; Cruces et al., 2014; Huang et al., 2015; Liang et al., 2019; Pereda-Pérez et al., 2019). It is believed that while short-term stressors induce adaptive responses, continued stress produces maladaptive changes in organisms. Prolonged exposure to high glucocorticoids levels alters the structure and function of neural regions implicated in learning and memory, particularly the hippocampus (McEwen and Sapolsky, 1995; McEwen, 1999; Sapolsky, 2003; Leuner and Gould, 2010; Mora et al., 2012), amygdala (Cahill and McGaugh, 1998; Berretta, 2005; Roozendaal and McGaugh, 2011; Mora et al., 2012), as well as the medial prefrontal and anterior cingulated cortices (Allman et al., 2001; Akirav and Maroun, 2007; Holmes and Wellman, 2009; Mora et al., 2012). Glucocorticoid hypersecretion is associated with reduced hippocampus volume and determined prefrontal cortex subregions, while the basolateral amygdala is hypertrophied. Therefore, chronic stress is commonly considered to impair learning and memory in hippocampal-dependent tasks, such as spatial learning, but to enhance performance in those tasks that are amygdala-dependent, such as auditory fear conditioning (Sandi and Pinelo-Nava, 2007). Previously, we showed that long-term isolation housing produced a deficit in contextual fear learning, an amygdala and hippocampus-dependent task, but not of auditory-cued fear memory in degus (Pereda-Pérez et al., 2013). Subsequently, we observed that individual housing resulted in a reduction of hippocampal synaptic PSA-NCAM content and the hippocampal CA1 subfield volume, without significant changes in the size of the CA3 subregion or the total hippocampus volume. However, these changes seem not to be essential for learning in allocentric spatial memory tasks such as the Barnes maze. Interestingly, in this sense, recent data indicated that the Barnes maze did not significantly change synaptic efficacy in the CA1 area of the dorsal hippocampus in rats (Sadeghian et al., 2019). Furthermore, *in vivo* electrophysiological studies have reported that spatial learning and memory are supposed to be mainly associated with synaptic plasticity in distinct parts of the trisynaptic circuit of the hippocampus. Thus, the acquisition of spatial learning when the animal explores visual landmarks has been found to induce plasticity in the perforant path to dentate gyrus or mossy fibers to CA3 synapses, but not in the CA1 area (Manahan-Vaughan and Braunewell, 1999; Kemp and Manahan-Vaughan, 2008; Hagena and Manahan-Vaughan, 2011). In addition, it has been suggested that chronic exposure to a high amount of sensory information causes a decrease in animals’ attention to salient and/or subtle environmental stimuli (Walasek et al., 2002). In the present study, individually housed female degus were not devoid of receiving sensory information from other peers through olfaction and hearing, even though barriers between the cages prevented them from visual access. It is possible that these degus were more aware of context signals which enable them to perform more efficiently in a spatial reference task such as the Barnes maze test, which is considered mildly aversive. Moreover, the present isolation protocol did not affect plasma cortisol levels, as the levels were similar in both the isolated and grouped-housed animals. In conclusion, the present study indicates that, in adult female degus, individual housing significantly improved the acquisition of spatial learning but slightly disrupted spatial retrieval memory.

## Data availability statement

The raw data supporting the conclusions of this article will be made available by the authors, without undue reservation.

## Ethics statement

The animal study was reviewed and approved by the European Communities Council Directive of 24 November 1986 (86/609/EEC) and the guidelines issued by the Spanish Ministry of Agriculture, Fishing and Feeding (Royal Decree 1201/2005 of 21 October 2005) and were approved by the Institutional Animal Ethics Committee (Reference No. A13160603). Efforts were made to minimize the number of animals used, as well as their suffering.

## Author contributions

MR and JM provided degus and animal facilities supporting. NP, BB-O, and MP performed the experiments. NP and MP analyzed the video records, managed the literature searches, and contributed to drafting the work. MR, JM, BB-O, and CV participated in writing, reviewing, and editing. MP undertook the statistical analysis and data presentations. MR, JM, and CV provided financial support. All authors participated in work planning and approved the final manuscript.
